# Acute Alcohol Intoxication Modulates Monocyte Subsets and Their Functions in a Time-Dependent Manner in Healthy Volunteers

**DOI:** 10.3389/fimmu.2021.652488

**Published:** 2021-05-18

**Authors:** Andrea Janicova, Florian Haag, Baolin Xu, Alejandra P. Garza, Ildiko Rita Dunay, Claudia Neunaber, Aleksander J. Nowak, Paola Cavalli, Ingo Marzi, Ramona Sturm, Borna Relja

**Affiliations:** ^1^ Experimental Radiology, Department of Radiology and Nuclear Medicine, Otto von Guericke University, Magdeburg, Germany; ^2^ Institute of Inflammation and Neurodegeneration, Otto-von-Guericke University Magdeburg, Magdeburg, Germany; ^3^ Trauma Department, Hannover Medical School, Hannover, Germany; ^4^ Department of Trauma, Hand and Reconstructive Surgery, Goethe University, Frankfurt, Germany

**Keywords:** ethanol, drinking, innate immunity, inflammasome, LPS, IL-1β, CD14, TLR4

## Abstract

**Background:**

Excessive alcohol intake is associated with adverse immune response-related effects, however, acute and chronic abuse differently modulate monocyte activation. In this study, we have evaluated the phenotypic and functional changes of monocytes in acutely intoxicated healthy volunteers (HV).

**Methods:**

Twenty-two HV consumed individually adjusted amounts of alcoholic beverages until reaching a blood alcohol level of 1‰ after 4h (T4). Peripheral blood was withdrawn before and 2h (T2), 4h (T4), 6h (T6), 24h (T24), and 48h (T48) after starting the experiment and stained for CD14, CD16 and TLR4. CD14^bright^CD16^-^, CD14^bright^CD16^+^ and CD14^dim^CD16^+^ monocyte subsets and their TLR4 expression were analyzed by flow cytometry. Inflammasome activation *via* caspase-1 in CD14^+^ monocytes was measured upon an *ex vivo in vitro* LPS stimulation. Systemic IL-1β and adhesion capacity of isolated CD14^+^ monocytes upon LPS stimulation were evaluated.

**Results:**

The percentage of CD14^+^ monocyte did not change following alcohol intoxication, whereas CD14^bright^CD16^-^ monocyte subset significantly increased at T2 and T24, CD14^bright^CD16^+^ at T2, T4 and T6 and CD14^dim^CD16^+^ at T4 and T6. The relative fraction of TLR4 expressing CD14^+^ monocytes as well as the density of TLR4 surface presentation increased at T2 and decreased at T48 significantly. TLR4^+^CD14^+^ monocytes were significantly enhanced in all subsets at T2. TLR4 expression significantly decreased in CD14^bright^CD16^-^ at T48, in CD14^bright^CD16^+^ at T24 and T48, increased in CD14^dim^CD16^+^ at T2. IL-1β release upon LPS stimulation decreased at T48, correlating with TLR4 receptor expression. Alcohol downregulated inflammasome activation following *ex vivo in vitro* stimulation with LPS between T2 and T48 *vs*. T0. The adhesion capacity of CD14^+^ monocytes decreased from T2 with significance at T4, T6 and T48. Following LPS administration, a significant reduction of adhesion was observed at T4 and T6.

**Conclusions:**

Alcohol intoxication immediately redistributes monocyte subsets toward the pro-inflammatory phenotype with their subsequent differentiation into the anti-inflammatory phenotype. This is paralleled by a significant functional depression, suggesting an alcohol-induced time-dependent hyporesponsiveness of monocytes to pathogenic triggers.

## Introduction

Alcohol is one of the oldest and nowadays one of the most common addictive substances worldwide (1). Although low-to-moderate alcohol intake is associated with decreased risk of cardiovascular diseases and subsequent mortality (2), alcohol abuse contributes to the development of alcoholic liver disease, pancreatitis and further pathologies (1). In Germany, approximately 7.8 million adults drink excessive amounts of alcohol (3), causing 74,000 deaths every year (4). However, these numbers do not include physical trauma-related deaths that frequently occur under the influence of alcohol (4). Since up to 50% of physically traumatized patients are admitted to the emergency departments with an acute alcohol intoxication (5, 6), the numbers of alcohol-related deaths may be underestimated.

There is a rising evidence that alcohol intake has a biphasic effect on the innate immune response in a dose- and time-dependent manner (7–10). Chronic alcohol intake is associated with increased susceptibility to infections and sterile inflammation, which may cause severe tissue damage and poor outcomes after injury (1, 11). Following binge drinking, defined as an episodic excessive alcohol intake and the most common form of alcohol abuse, the counts of circulating leukocytes, monocytes and natural killer cells as well as the release of tumor necrosis factor (TNF)-α after an *ex vivo in vitro* whole blood stimulation with lipopolysaccharide (LPS) increase in the first 20 minutes after drinking, suggesting an early pro-inflammatory response. Subsequent decline of monocytes, natural killer cells and interleukin (IL)-1β, IL-6 and monocyte chemoattractant protein (MCP)-1 levels in circulation and the elevation of systemic IL-10 level suggest an anti-inflammatory state in the later course (7, 9, 12). In line with this data, leukocytes and IL-6 decrease in circulation was observed in severely injured patients with excessive alcohol intoxication (10). However, lower alcohol concentrations do not seem to induce the pro-inflammatory acute phase proteins (9). Those pro-inflammatory mediators are essential for the cell recruitment of the innate immune system and the initiation of the adaptive immune response (8), why acutely intoxicated patients with alcohol are potentially more vulnerable to infections.

Human monocytes exposed to moderate amount of alcohol *in vivo* or *in vitro* express significantly lower levels of pro-inflammatory cytokines in Toll-like receptor (TLR)4-MyD88 and TLR-TRIF-dependent manner when stimulated with LPS *in vitro* (13). Additionally, activation of the nuclear factor 'kappa-light-chain-enhancer' of activated B-cells (NF-κB) is downregulated p65/p50 dependently (14). The above described studies deal with monocytes as a single population, however, three monocyte subsets can be distinguished according to their expression levels of cluster of differentiation (CD)14 and CD16: Classical monocytes (CD14^bright^CD16^-^) are released into circulation from bone marrow and act as a precursor for intermediate monocytes (CD14^bright^CD16^+^), which in turn differentiate into non-classical monocytes (CD14^dim^CD16^+^) (15). Classical subset has anti-microbial and innate immune sensing features, contributing to phagocytosis, adhesion and migration (15). Once they enter tissues, classical monocytes differentiate into monocyte-derived macrophages or dendritic cells, where they are crucial for shaping and subsequent resolution of inflammation (15). Intermediate monocytes regulate apoptosis and transendothelial migration (15), and their high occurrence in patients with systemic infections suggests a defensive role against invading pathogens (16). Non-classical monocytes provide complement- and FcR-mediated phagocytosis and are linked with anti-viral response to human immunodeficiency virus (15). In chronic excessive alcohol abuse, the numbers of classical monocytes are significantly reduced, whereas the non-classical monocytes display an increase in their counts, while the changes particularly restore two weeks following alcohol withdrawal (17). Although accumulating evidence indicates a shift in monocyte subsets under several pathological conditions, their distribution and function in acutely intoxicated subjects with alcohol remain elusive.

Considering that phenotypic and functional alterations of monocytes are substantially involved in the initiation and subsequent resolution of inflammation, we evaluated the phenotypic shift of monocytes following episodic excessive alcohol intake in healthy volunteers (HV). We hypothesized that acute alcohol intoxication shifts the monocytes toward the anti-inflammatory phenotype and ameliorates LPS-induced pro-inflammatory response *ex vivo in vitro* by suppressing the inflammasome activation.

## Materials and Methods

### Ethics

The current study was performed pursuant to ethics committee approval (255/14) from the University Hospital of the Goethe-University Frankfurt and in accordance with the Declaration of Helsinki and following the Strengthening the Reporting of Observational studies in Epidemiology-guidelines (18).

### Patient Cohort

Twenty-two HV between 18 and 50 years of age were enrolled. Exclusion criteria included a history of chronic alcohol abuse, acute infection, pre-existing chronic inflammatory diseases, immunological disorders, human immunodeficiency virus infection, infectious hepatitis, medication and pregnancy. Renal insufficiency and changes in transaminases to identifying chronic liver diseases were excluded by blood test.

### Study Design

**Figure 1:**
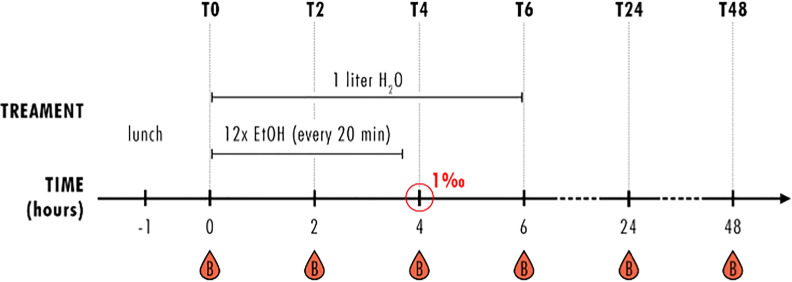
Study design. Healthy volunteers (HV) received a standardized lunch one hour before starting the experiment and had one liter water at hand during the first observation period of 6 hours (T0-T6). Every 20 minutes, HV received an individually calculated amount of whisky-coke cocktail (EtOH) for four hours, reaching blood alcohol concentration of 1‰ after 4 hours (T4). Blood samples were collected before (T0) and 2 (T2), 4 (T4), 6 (T6), 24 (T24) and 48 (T48) hours after starting the alcohol consumption. This is the same study design as published by Sturm et al. (19).

Healthy volunteers received a standardized lunch (1 pizza) one hour before starting the experiment. Subsequently, HV consumed individually calculated amount of alcohol until reaching blood alcohol level of 1 ‰ after 4 hours. In detail, HV received whisky-coke cocktail (Tennessee Whiskey Jack Daniels, 40%; Coca-Cola) in a mixing ratio of 1:2 every 20 minutes for 4 hours. Then, the HV were monitored for further 2 hours without alcohol intake. For the individual calculation of the amount of alcohol to reach 1 ‰ after 4 hours, the modified Widmark equation including age, sex, height and weight was applied. Blood alcohol concentration (BAC) was monitored hourly until T6 and at T24 and T48. At every time point, a blood sample for the determination of the BAC was taken. Blood samples were obtained in prechilled ethylenediaminetetraacetic acid tubes (BD vacutainer; Becton Dickinson Diagnostics, Belgium) and kept on ice. Then, blood was centrifuged at 2,000 × g and at 4°C for 15 minutes and the supernatant was immediately used for the determination of BAC using the diagnostic set serum EtOH by Cobas 8000 Modular Analyzer (Roche Diagnostic, Germany). The participants leaved the research facility after T6 and came back at T24 and T48. During the time out of the institute, participants were allowed to eat and drink with restriction of drinking alcohol. A brief overview of the study design and sampling is shown in **Figure 1**.

### Blood Sampling

Peripheral blood was withdrawn in serum-gel and Li-Heparin collection tubes (Sarstedt, Germany) before (T0) and 2 (T2), 4 (T4), 6 (T6), 24 (T24) and 48 (T48) hours after starting the alcohol consumption. Subsequently, the samples collected in serum-gel collection tubes were centrifuged directly after the collection at 2,000 × g and at 4°C for 15 minutes. Serum was stored at -80°C until further analysis. Blood in Li-Heparin collection tubes were directly processed for flow cytometry and adhesion assay.

### Analysis of Monocyte Subsets and TLR4 Expression by Flow Cytometry

100 µl heparinized blood was transferred into polystyrene FACS tubes (BD Pharmingen™). The samples were incubated with 5 µl CD14 (APC/Cy7; Clone M5E2; BioLegend), 5 µl CD16 (PE/Cy7; Clone 3G8; BioLegend) and 5 µl TLR4 (APC; Clone HTA125; BioLegend) in the dark at 4°C for 10 minutes. Subsequently, the samples were washed with 2 ml FACS buffer (0.5% bovine serum albumin (BSA) in phosphate-buffered saline (PBS)) and centrifuged at 350 × g at 4°C for 5 minutes. For red blood cells lysis, 3 ml lysis buffer (0.155 M NH_4_Cl, 0.01 M KHCO_3_, 0.1 mM ethylenediaminetetraacetic acid (EDTA)) were added and incubated in the dark at 4°C. Washing step was repeated. Monocyte population was defined by gating the corresponding forward and side scatter scan. From each sample a minimum of 3.0 x 10^4^ cells were measured, which were subsequently analyzed. The percentage of CD14^bright^CD16^-^ (classical subset), CD14^bright^CD16^+^ (intermediate subset) and CD14^dim^CD16^+^ (non-classical subset) as well the percentage and mean fluorescent units of TLR4 out of CD14^+^ viable cells were assessed by flow cytometric analyses using a BD FACS Canto 2™ and FACS DIVA™ software (BD Bioscience). Unspecific binding of the antibodies was excluded by using isotype controls.

### Whole Blood Stimulation With LPS

50 µl heparinized blood was added to 450 µl RPMI 1640 media with supplements [100 UI/ml penicillin, 100 µg/ml streptomycin, 0.1 mg/ml gentamycin, 20 mM 4-(2-hydroxyethyl)-1-piperazineethanesulfonic acid, 10% heat-inactivated fetal bovine serum (FBS)] and LPS at final concentration of 10 µg/ml (20). Subsequently, the samples were incubated at 37°C and 5% CO_2_ for 24 hours. After stimulation, the samples were centrifuged at 2,100 × g and at room temperature for 10 minutes. Supernatants were stored at -80°C until the further analysis.

### Measurement of IL-1β 

For the detection of IL-1β levels in directly collected serum (see Blood Sampling) and the supernatants after whole blood stimulation with LPS (see Whole Blood Stimulation With LPS), ELISA DuoSet kit (#DY201, R&D, USA) was used according to the provider's instructions.

### Caspase-1 Activity

Active caspase-1 was quantified by using a FAM-FLICA Caspase-1 (YVAD) Assay Kit (ImmunoChemistry, USA) (21). 50 µl blood and 240 µl RPMI 1640 media with supplements was transferred into polystyrene FACS tubes. Samples were incubated with 1 µg/ml LPS (*E. coli* O127:B8 strain; Sigma Aldrich, Germany), 100 µM BzATP (ATP; Sigma-Aldrich, Germany) (22) and 10 µl 30X FAM-FLICA caspase-1 inhibitor) at 37°C and 5% CO_2_ for 90 minutes. Subsequently, cells were washed with RPMI 1640 media supplemented with 10% FBS 2 times. For red blood cell lysis, 3 ml lysis buffer (0.155 M NH_4_Cl, 0.01 M KHCO_3_, 0.1 mM EDTA) were added and incubated in the dark at 4°C for 10 minutes. Cells were washed with 2 ml FACS buffer and centrifuged at 200 × g at 4°C for 5 minutes. Following pellet resuspension in 500 µl FACS buffer, caspase-1 expression was assessed by flow cytometric analyses using a BD FACS Canto 2™ and FACS DIVA™ software (BD).

### Adhesion Assay

The isolation of CD14^+^ monocyte we described already elsewhere (23). Peripheral blood monocytes were isolated by a density-gradient centrifugation (Bicoll separation solution, 1.077 g/ml density; Biochrom, Germany). Here, 20 ml Bicoll separating solution was carefully overlaid with 20 ml of heparinized blood and centrifuged at 800 × g at room temperature for 30 minutes. Peripheral blood mononuclear cells from interphase were transferred into fresh tubes and washed with 10 ml FACS buffer and centrifuged at 350 × g at room temperature for 5 minutes. Remaining red blood cells were lysed by 10 ml lysis buffer in dark for 10 minutes and subsequently centrifuged. Following a further washing step with 15 ml MACS buffer (0.5% BSA, 2 mM EDTA in 1x PBS w/o Mg^2+^ and Ca^2+^), cells were resuspended in 500 µl RPMI 1640 media with supplements. Mononuclear cells were stimulated with 1 µg/ml LPS at 37°C and 5% CO_2_ for 90 minutes and subsequently washed with MACS buffer.

For CD14 labeling, cell pellet was resuspended in 75 µl MACS buffer and the cell suspension was incubated with 25 µl magnetic CD14 microbeads (Miltenyi Biotec, Germany) at room temperature for 15 minutes. Following a washing step with 3 ml MACS buffer, CD14^+^ labeled monocytes were magnetically isolated by using LS columns (Miltenyi Biotec, Germany) according to the manufacturer’s protocol. Following a further washing step with 10 ml FACS buffer, cell pellet was resuspended in 500 µl of RPMI 1640 media with supplements. Only cell cultures with a purity and viability of ≥95% were used for further experiments. Cell population purity was evaluated by flow cytometry according its size and CD14 expression. Cell viability was proved by Türk’s solution exclusion assay (Merck, Darmstadt, Germany).

The adhesion assay was performed as described before (24). 200,000 CD14^+^ monocytes were seeded in 6-well flat-bottom plate (Sarstedt, Germany), where adherent A549 pulmonary epithelial cells to the density of 80% were seeded a day before, and, incubated at 37°C and 5% CO_2_ for 35 minutes. Cells were washed twice with RPMI 1640 media with supplements and following fixation by 1 ml 1% glutaraldehyde for 3 minutes, monocytes were washed once more with PBS. Adherent monocytes on plates were stored in 2 ml PBS in the dark and at 4°C until evaluation. The adherent monocytes were then counted in 5 different fields of a defined size (5×0.25 mm^2^) using a phase contrast microscope (×20 objective). The mean cellular adhesion rate was calculated.

### Statistics

GraphPad Prism 6.0 software (GraphPad Software Inc. San Diego, CA, USA) was used to perform the statistical analysis. Data are given as mean ± standard error of the mean (SEM). The data distribution was tested by the D’Agostino-Pearson test, and the data is not normally distributed. Since we have 6 groups with matched or repeated measures, thus paired data, the non-parametric Friedman’s test was applied. Dunn-Bonferroni post-hoc test for multiple comparisons was used. T0 is the base condition to which the others were compared. The Wilcoxon matched-pairs signed rank test as a nonparametric statistical tool comparing two paired groups in **Figure 6** was used to determine if two sets of pairs are different from one another in a statistically significant manner. Spearman’s correlation coefficient was calculated to determine correlations. A p value below 0.05 was considered statistically significant.

## Results

### Characteristics of the Study Participants

From 22 healthy volunteers, 12 female and ten male with a mean age of 25 (± 4) years were enrolled. All included individuals exerted normal ranges of transaminases excluding chronic e.g. liver diseases, since the rages at T0 were: 15-20 U/l GGT, 22 U/l GPT and 23 U/l GOT. Thus, no individuals with chronic liver diseases or malfunction were included. Furthermore, the BAC increased to 0.46±0.02‰ at T2, with further at T4 (1.11±0.05‰) reaching the target BAC of 1‰. At T6 BAC decreased to 0.83±0.06‰ and was not further detectable at T24 and T48.

### Acute Alcohol Intake Modulates the Distribution the CD14^+^ Monocyte Subsets

**Figure 2:**
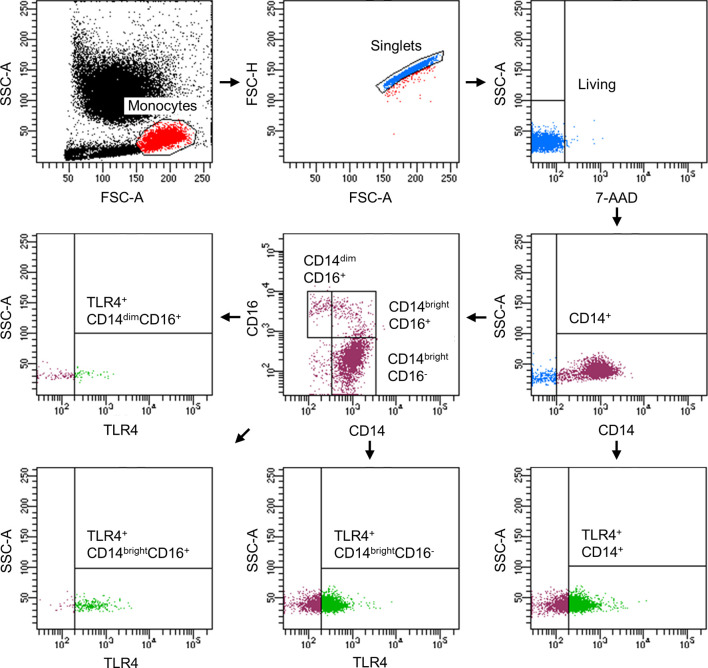
Representative gating strategy for the flow cytometric analyses and evaluation of monocyte subsets with regard to their TLR4 expression.

**Figure 3:**
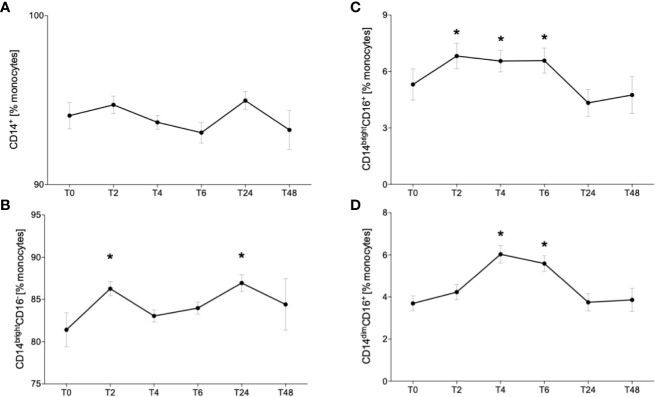
Impact of acute alcohol intoxication on monocyte subset distribution. Percentage distribution of CD14^+^ **(A)** classical (CD14^bright^CD16^-^) **(B)**, intermediate (CD14^bright^CD16^+^) **(C)** and non-classical (CD14^dim^CD16^+^) **(D)** was determined in blood of healthy volunteers (n=22) before (T0) and 2 (T2), 4 (T4), 6 (T6), 24 (T24) and 48 (T48) hours after starting the alcohol consumption. Data are presented as mean ± standard error of the mean. *p < 0.05 *vs*. T0.

Since it is known that alcohol misuse has modulating effects on the immune system, we investigated the distribution of different monocytes subsets in healthy volunteers following excessive acute alcohol intake. Fluorescently *ex vivo in vitro* labeled circulating monocytes were gated as it is shown in the **Figure 2**. Following alcohol intake, the percentage of CD14^+^ monocytes did not change during the whole observational period of 48 hours compared to the counts before the experiment start (T0) (**Figure 3A**). Regarding the subsets, the classical monocytes defined as CD14^bright^CD16^-^ cells became significantly more abundant at T2 and T24 after starting drinking alcohol compared to T0 (**Figure 3B**, p < 0.05). The intermediate CD14^bright^CD16^+^ monocytes displayed a significant increase in the first 6 hours (T2, T4, T6) with subsequent decrease to the baseline at T24 and T48 (**Figure 3C**, p < 0.05). We observed an increase of non-classical CD14^dim^CD16^+^ subset at T4 and T6 hours compared to the counts at T0, followed by decrease from T24 (**Figure 3D**, p < 0.05).

Combining the absolute cell numbers of leukocytes per microliter (25) with the relative fractions of monocytes and their subsets, we converted the percentages of monocyte subsets into absolute cell numbers. These are summarized in **Supplementary Table 1**.

### TLR4 Expression Is Upregulated in the Early Phase and Downregulated in the Later Phase of Acute Alcohol Intake

**Figure 4:**
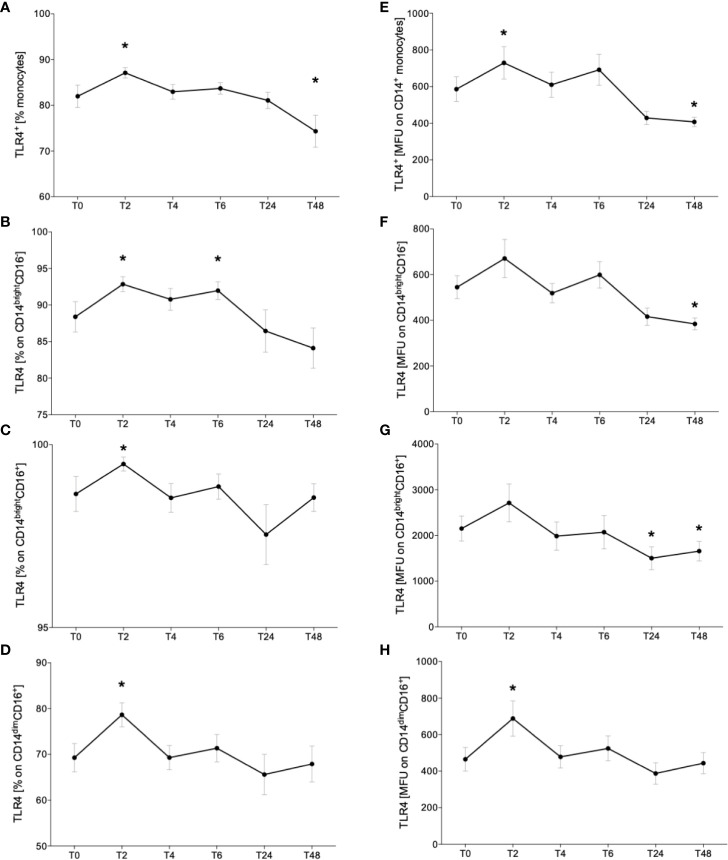
Impact of acute alcohol intoxication on Toll-like receptor 4 (TLR4) surface presentation on monocytes. Percentage and mean intensity expression of TLR4 on CD14^+^ monocytes **(A, E)** and the classical (CD14^bright^CD16^-^) **(B, F)**, intermediate (CD14^bright^CD16^+^) **(C, G)** and non-classical (CD14^dim^CD16^+^) **(D, H)** monocyte subsets were determined in blood of healthy volunteers (n=22) before (T0) and 2 (T2), 4 (T4), 6 (T6), 24 (T24) and 48 (T48) hours after starting the alcohol consumption. Data are presented as mean ± standard error of the mean. *p < 0.05 *vs*. T0.

Following the determination of monocyte subsets, we analyzed the expression of surface receptor TLR4. The representative gating strategy of TLR4-positive monocytes is shown in the **Figure 2**. The abundance of TLR^+^ CD14^+^ monocytes significantly increased at T2 while decreasing to base level at T6 until T24 (**Figure 4A**, p < 0.05). At T48, TLR4 expression was significantly decreased compared to T0 (**Figure 4A**, p < 0.05). The relative fraction of TLR4^+^ CD14^bright^CD16^-^ classical monocytes increased significantly at T2 and T6 *vs*. T0 (**Figure 4B**, p < 0.05). CD14^bright^CD16^+^ intermediate and CD14^dim^CD16^+^ non-classical subsets showed a significant elevation of TLR4^+^ monocytes at T2 compared to T0 (**Figures 4C, D**, p < 0.05).

Additionally, to the relative fraction of TLR4^+^ monocytes, we evaluated the mean TLR4 expression on monocytes. CD14^+^ monocytes displayed significantly higher expression at T2 and significantly reduced TLR4 receptor density at T48 compared to T0 (**Figure 4E**, p < 0.05). Rising trend was indicated at T2 and T6 in classical monocyte subset and the density of TLR4 decreased significantly at T24 *vs*. T0 (**Figure 4F**, p < 0.05). Intermediate monocytes showed increased TLR4 expression at T2 and a significant decline at T24 and T48 compared to T0 (**Figure 4G**, p < 0.05). The non-classical subset displayed significant elevation of TLR4 receptor intensity at T2 *vs*. T0 (**Figure 4H**, p < 0.05).

### IL-1β Release Following *Ex Vivo In Vitro* Whole Blood Stimulation With LPS Is Diminished Two Days After Acute Alcohol Intake Correlating With TLR4 Receptor Density on Monocytes

**Figure 5:**
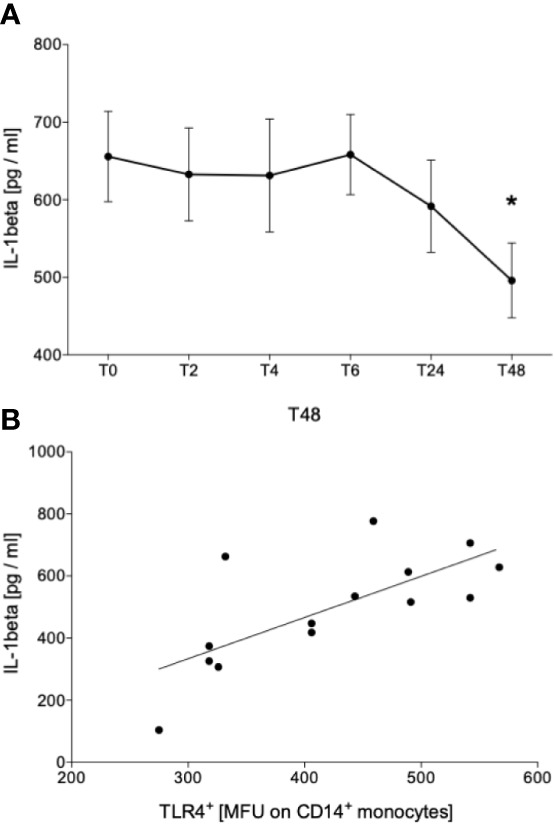
Impact of acute alcohol intoxication on IL-1β release after stimulation with lipopolysaccharide (LPS). IL-1β was determined in blood of healthy volunteers (n=22) that was stimulated with LPS for 24 h before (T0) and 2 (T2), 4 (T4), 6 (T6), 24 (T24) and 48 (T48) hours after starting the alcohol consumption **(A)**. Spearman analysis of correlation between IL-1β and TLR4 at T48 is shown **(B)**. Data are presented as mean ± standard error of the mean. *p < 0.05 *vs*. T0.

Further, since it is known that ethanol inhibits the NLRP3 inflammasome activation, we evaluated the levels of circulating IL-1β and following *ex vivo in vitro* stimulation with LPS. IL-1β was not detectable in serum directly obtained from the participants at all time points (detection limit of 3.91 pg/mL; data not shown). Following LPS stimulation, we have shown that the acute ethanol intoxication did not have an impact on IL-1β concentration at T2, T4 and T6 compared to IL-1β concentration before starting binge drinking (T0) (**Figure 5A**). At T24, we observed a slight decrease that continued and became significant at T48 *vs*. T0 (**Figure 5A**, p < 0.05). This IL-1β level positively correlated with TLR4 receptor density on monocytes at T48 (**Figure 5B**).

### Ethanol Intake Leads to Hyporesponsiveness to Secondary Hit

**Figure 6:**
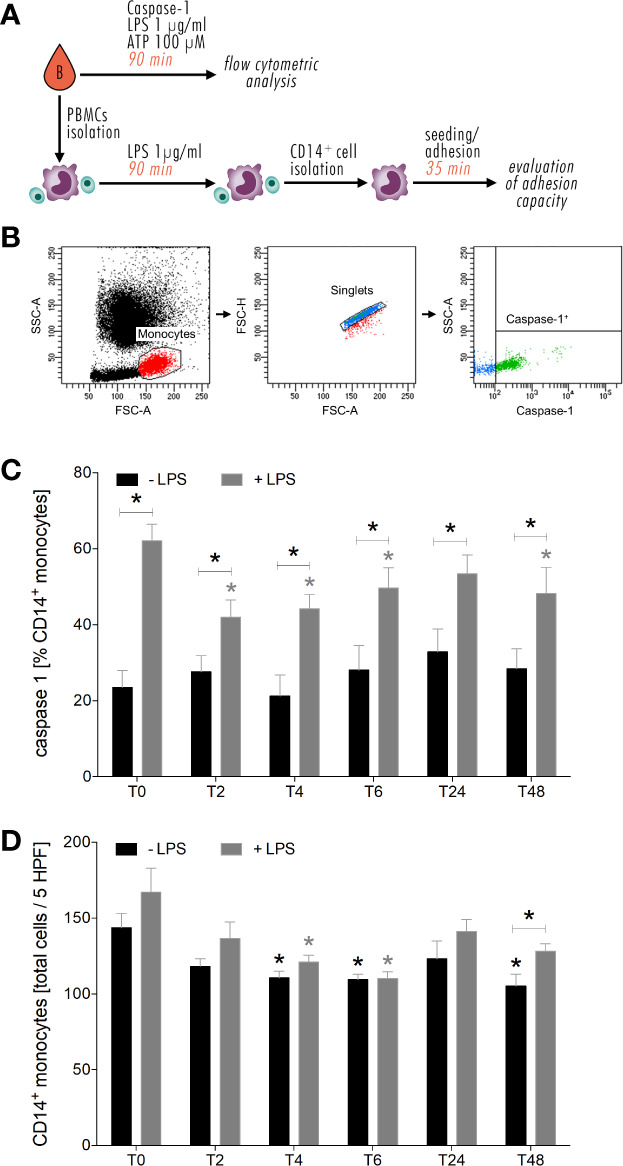
Impact of acute alcohol intoxication on inflammasome activation in monocytes and their adhesive capacity upon LPS and ATP or only LPS stimulation *ex vivo*. Experimental design and representative gating strategy for caspase-1 is shown in **(A, B)**. Percentage expression of caspase-1 in CD14^+^ monocytes **(C)** and their adhesive capacity **(D)** following *ex vivo* LPS stimulation was determined in blood of healthy volunteers (n=22) before (T0) and 2 (T2), 4 (T4), 6 (T6), 24 (T24) and 48 (T48) hours after starting the alcohol consumption. Data are presented as mean ± standard error of the mean. *p < 0.05 *vs*. T0 and indicated.

A brief overview about *ex vivo in vitro* experimental design and representative gating strategy is shown in **Figures 6A**, **B**. Level of active caspase-1 did not show significant changes in CD14^+^ monocytes during the entire observation period of 48 hours compared to the level before alcohol intoxication (T0) (**Figure 6C**). The *ex vivo in vitro* stimulation of CD14^+^ monocytes with 1 µg/mL LPS and 100 µM ATP led to significant increase of caspase-1 expression at all time points compared to equivalent unstimulated monocytes (**Figure 6C**, p < 0.05). At T2, T6 and T48, caspase-1 expression significantly decreased following LPS and ATP stimulation compared to stimulated monocytes at T0 (**Figure 6C**, p < 0.05).

Furthermore, we found that the adhesion capacity of CD14^+^ monocytes is impaired by alcohol intake. *Ex vivo in vitro*, CD14^+^ monocytes displayed less adhesion to A549 cells from T2 until T48 compared to T0, with significance at T4, T6 and T48 (**Figure 6D**, p < 0.05). Following LPS administration, monocyte adhesion decreased from T6 until T48 *vs*. stimulated monocytes at T0, with significances at T4 and T6 (**Figure 6D**, p < 0.05). At T48, significant increase in adhesion rates of LPS stimulated monocytes compared to unstimulated monocytes is shown (**Figure 6D**, p < 0.05). At T6, there is no difference in adhesion rates between stimulated und unstimulated monocytes (**Figure 6D**).

## Discussion

**Figure 7:**
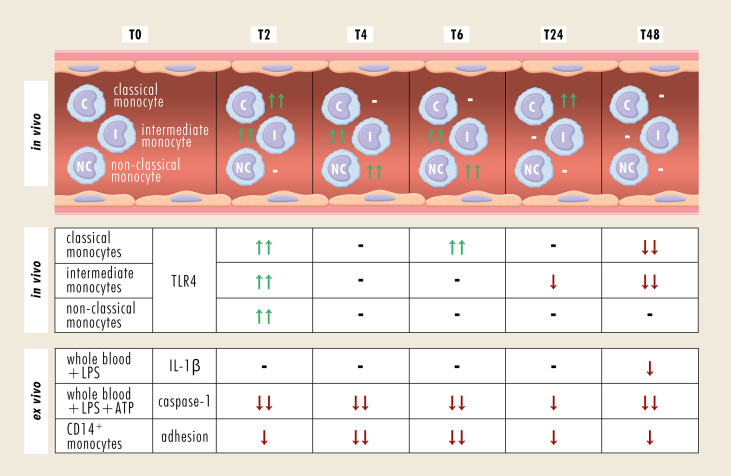
Summary of phenotypical and functional changes of monocyte subsets following an acute alcohol intoxication. -, no change; ↑↑, significant increase; ↓↓, significant decrease; ↓, decreasing trend; ATP, adenosine triphosphate; CD, cluster of differentiation; IL-1β, Interleukine-1β; LPS, lipopolysaccharide; TLR4, Toll-like receptor 4.

Excessive alcohol drinking is associated with adverse immune response-related effects such as susceptibility to nosocomial infections with possible further progression to acute respiratory distress syndrome or sepsis (26). Following traumatic injury, monocytes show a shift toward the pro-inflammatory phenotype (23), and whereas chronic alcohol intake causes an exaggerated differentiation into the anti-inflammatory phenotype (17), the effect of acute alcohol intake still remains elusive. Since there is a frequent coincidence of alcohol misuse and traumatic injuries (5, 6), understanding of their interaction and the mechanisms of ongoing misbalanced immune response after trauma may contribute to the development of advanced therapeutic strategies for alcohol-intoxicated severely injured patients. Therefore, we evaluated the phenotypic and functional changes of monocytes in intoxicated HV. The key results are summarized in **Figure 7**.

In this study, HV consumed calculated amount of whisky-coke cocktail every 20 minutes during the first 4 hours of the experiment, reaching a blood alcohol level of 1‰. Over the whole observation period of 48 hours, the relative fraction of CD14^+^ monocytes remained stable, whereas the individual monocyte subsets showed significant alterations. Classical monocytes, characterized by CD14^bright^CD16^-^ expression, provide phagocytosis contributing to immune defense against invading pathogens and are equated with murine pro-inflammatory Ly6C^bright^ monocytes (15, 27). Two hours after drinking, the ratio of classical subset significantly increased. This was paralleled by increased abundance of intermediate subset that lasted for 6 hours and by enhanced levels of non-classical monocytes between 4 and 6 hours after alcohol intake. It is generally accepted that classical monocytes are precursors for the intermediate subset, which expresses the highest levels of antigen presentation-related molecules and has been linked with rapid immune defense in systemic infections (15, 16). Alcohol intoxication has been shown to enhance the expression of MCP-1 and C-C chemokine receptor type 2 in pancreas and microglia, contributing to the extent of tissue injury (28, 29). Therefore, binge alcohol drinking may cause a release of classical monocytes from the bone marrow within the first 2 hours after drinking, which in turn may differentiate into intermediate monocytes. Regarding the delayed increase of the relative non-classical monocyte fraction, it seems, that alcohol intoxication converts the intermediate into non-classical monocytes and thereby, switch their pro-inflammatory character into an immunosuppressive cell type. Under the same experimental settings, it has been shown that absolute leukocyte numbers significantly increase two and four hours after starting alcohol drinking, suggesting an early mobilization of immune system (25). The percentage of granulocytes out of all leukocytes decreases significantly at four and six hours, whereas the monocyte percentage decreases first significantly at six hours with subsequent significant elevation at 24 and 48 hours post-drinking (25). The current literature describes changes in leukocyte subpopulations almost exclusively in chronic alcohol abuse, although these changes are not less relevant in acute setting of alcohol drinking. Gacouin et al. analyzed leukocytes in critically ill patients, which have been acutely intoxicated with alcohol (30). They have shown that the circulating neutrophils and classical monocytes are less present in those patients, whereas the values for B lymphocytes, cytotoxic and noncytotoxic lymphocytes were significantly higher. Since these results persist in patients with and without infection, they suggest that these changes may be induced by alcohol.

The initial pro-inflammatory immune response is paralleled by elevated TLR4 expression 2 hours after starting the alcohol intake. Following an alcohol binge, the intestinal barrier is disrupted, enhancing the mucosal penetration of intestinal luminal toxic substances, pathogens and antigens, that in turn can lead to intestinal mucosal injury and inflammation (31). An increase of TLR4 in ileum and colon has been linked with increased intercellular permeability by the disruption of tight junctions between epithelial cells (32). Alcohol-induced loss of tight junctions allows the intestinal pathogens or pathogen-derived molecules to translocate into circulation, where they trigger the inflammatory cascade and activate immune cells such as monocytes and macrophages in TLR4-dependent manner, leading to a secretion of pro-inflammatory cytokines (31–33). Moreover, the intensity of alcohol intoxication positively correlates with serum levels of LPS and markers of monocyte activation (33). However, 24 hours after binge drinking, we have shown that the TLR4 expression on monocytes is downregulated and positively correlates with systemic IL-1β levels measured at 48 hours following *ex vivo in vitro* LPS stimulation. The relative fraction of non-classical monocytes, which are comparable with murine anti-inflammatory Ly6C^low^ monocytes (27), recovered to the baseline at 24 hours. However, the immunosuppressive effect of alcohol seems to affect the functionality of individual monocyte subsets without changing their ratios at T48. In line with this, less monocytes are positive for reactive oxygen species at 48 hours after binge drinking, suggesting lower antimicrobial competence (25). Acute alcohol intoxication-related decrease in TLR4 response, so-called TLR4 tolerance, is induced by B-cell lymphoma 3-encoded protein (Bcl-3) that negatively regulates TNF-α transcription (34). Treatment of human monocytes and murine macrophages with 25 mM and 50 mM ethanol, respectively induces Bcl-3 expression that in turn enhances p50 homodimer stabilization. Bcl-3-p50 homodimer complex binds to TNF-α promotor region, inhibiting the TNF-α and consequently the NF-κB transcriptional activity (34, 35). However, repeated alcohol intoxication such as chronic alcohol abuse abolishes the initial anti-inflammatory effect and leads to the loss of TLR4 tolerance (1), supporting the theory about the biphasic effect of alcohol.

We have shown that systemic IL-1β level is below the detection limit following alcohol intoxication and that the amount of active caspase-1 does not significantly change in monocytes during the entire observation period. The release of IL-1β upon inflammasome activation involves 2 major steps: 1) Synthesis of inactive pro-IL-1β, 2) enzymatic cleavage of pro-IL-1β into active IL-1β by caspase-1 with subsequent secretion of IL-1β into extracellular space (36). Since LPS-induced IL-1β decrease positively correlates with TLR4 surface presentation at T48, monocytes appear less responsible toward TLR4 ligands such as LPS and may mitigate the transcription and translation of pro-IL-1β in NF-κB-dependent manner. No alteration in caspase-1 levels suggests that inflammasome assembly was not impeded and the lower IL-1β may be caused by the lack of inactive pro-IL-1β. Although it is well known that IL-1β is mainly produced by monocytic immune cells in NLR family pyrin domain containing 3 (NLRP3)/Caspase-1-dependent manner, there is increasing evidence that IL-1β can be produced by neutrophils or inflammasome-independently as well (37–39). Thus, a monocyte-independent production of IL-1β following alcohol intoxication should be further investigated.

*Ex vivo in vitro* stimulation of monocytes with LPS and ATP significantly increases their caspase-1 level. During and following the excessive alcohol intake, caspase-1 expression is upregulated but significantly less induced compared to non-intoxicated HV. This is in line with an *in vitro* study, that we have published recently showing that ethanol pre-treatment (170 mM) of human HepG2 liver cells downregulated caspase-1 expression following LPS and ATP administration (22). This may be caused by innate immune memory (40), whereby the first hit (here alcohol) may reprogram the innate immune cells resulting in an adaptation of the response to the subsequent second hit (here LPS and ATP). However, the underlying mechanism remains elusive. Some studies show that this adaptation may be caused by the downregulation of TLR surface expression and an upregulation of Toll-interacting protein with subsequent hypophosphorylation of IL-1 receptor-associated kinase and downregulation of canonical NF-κB pathway (41, 42). Moreover, acute alcohol intoxication leads to impaired adhesion of monocytes, whereby the subsequent *ex vivo in vitro* stimulation with LPS cannot at all or only barely increase the adhesive capacity compared to monocytes without endotoxin challenge. This may be caused by the lower surface presentation of intercellular adhesion molecule-1 and vascular cell adhesion molecule-1 as shown in an *in vitro* study with polyphenols from red wine by Ferrero et al. (43). Polyphenols are non-alcoholic compounds of alcoholic beverages such as wine or whisky (44). Since de-alcoholized wine inhibits the monocyte migration *ex vivo* (45), we should consider that the effect we attribute to ethanol/alcohol may be also caused by other non-alcoholic compounds.

### Limitations

This study has several strengths including pharmacologically relevant model for binge drinking and a novel approach to understanding the variability in monocyte subsets and the dynamic responses of these subsets as well as their functions following acute alcohol intoxication. However, this study also has several limitations. First, although we have shown a clear phenotypical shift of monocytes toward the anti-inflammatory phenotype in time-dependent manner, an analysis of the expression pattern of pro- and anti-inflammatory genes and of the altered functionality (e.g. phagocytosis and bacterial killing, generation of reactive oxygen species) would significantly improve these findings. This was not possible for technical and time reasons. Second, including a placebo group would immensely enhance the validity of the results. Until now, there are no short-term *in vivo* studies showing the impact of cola or its ingredients (sugar, phosphoric acid, carbonic acid etc.) on monocytes and the most *in vitro* studies focus on the long-term impact of glucose on monocyte functionality (diabetes models). Afshar et al. have shown in line with the data presented in this study that acute alcohol intoxication by ethanol mixed with chilled sugar-free flavored seltzer water led an early pro-inflammatory immune response 20 minutes after reaching blood alcohol concentration of 1‰ (7). Two and five hours later, an anti-inflammatory state was observed with reduced numbers of monocyte and natural killer cells, attenuated LPS-induced IL-1β levels and a trend toward increased IL-10 levels (7). Further, *in vitro* studies with THP-1 describe increased secretion of IL-1β in glucose dose- and time-dependent manner, that is paralleled by enhanced adhesion capacity (46, 47). Although these opposite results to our study cannot definitely exclude that cola might have anti-inflammatory features on monocyte subsets and functions, it considerably supports our conclusion that these effects are attributed to alcohol. Third, the calculated absolute cell numbers of monocyte subsets are artificial. Since we did not use cell counting beads, we obtained only the relative fractions of monocyte subsets. Thus, for getting the absolute cell numbers, we calculated those out of leukocytes counted by automated blood cell counter (Sysmex Europe GmbH, Germany). Lastly, although density gradient centrifugation by using Bicoll as a separation medium is a standard method for immune cell isolation for diverse immunological investigations, such as evaluation of the inflammasome activation in monocytes (48, 49), we cannot exclude a monocyte activation by the density gradient centrifugation itself or by direct or indirect interactions with lymphocytes and platelets, which have been shown to undergo phenotypical and functional changes following a density gradient centrifugation (50–52).

## Conclusions

Taking together, an acute intoxication with alcohol induces monocyte conversion toward their pro-inflammatory phenotype in the very early phase upon drinking. In the later time course, they differentiate into anti-inflammatory monocytes, findings that are paralleled by downregulation of TLR4 expression and IL-1β release. Our results suggest that an acute intoxication with alcohol has immunosuppressive effects in a time-dependent manner. Therefore, along with the endotoxin tolerance, excessively alcoholized subjects may be susceptible to the development of secondary infections. *Ex vivo in vitro* LPS stimulation of monocytes shows a downregulation of NLRP3 expression in samples obtained from severely injured patients compared to healthy subjects (53). This in turn elicits the synthesis and release of active IL-1β (53), whereby the monocyte deactivation correlates with the injury severity (54). In a rat model of blunt chest thorax trauma and hemorrhagic shock with subsequent resuscitation, monocytes express lower levels of caspase-1 in response to ethanol administration 2 hours before injury (21). Accordingly, alcohol intoxication may synergize with later trauma-induced immunosuppression leading to further enhanced vulnerability to infectious complications in the clinical course and has to be elaborated in further studies. It remains controversial whether an acute intoxication with alcohol has an impact on the outcome after a major injury, however, an adjustment of the timing of life-saving intervention according to the immune response may improve the therapeutic approach.

## Data Availability Statement

The original contributions presented in the study are included in the article/**Supplementary Material**. Further inquiries can be directed to the corresponding author.

## Ethics Statement

The studies involving human participants were reviewed and approved by ethics committee approval (255/14) from the University Hospital of the Goethe-University Frankfurt. The patients/participants provided their written informed consent to participate in this study.

## Author Contributions

BR designed the study, obtained the ethical approval, performed statistical analyses, and revised the manuscript. RS, FH, BX, and AJ collected the samples and performed the experiments. AJ and RS performed the statistical analyses, and AJ wrote the original draft. AG, ID, CN, AN, and PC contributed to analyses and revised the manuscript. ID, CN, and IM contributed intellectually to the completion of the study. All authors contributed to the article and approved the submitted version.

## Funding

The study was supported by grants from the Deutsche Forschungsgemeinschaft (grant nos. DFG RE 3304/5-1, DFG RE 3304/9-1, DFG NE 1932/1-3 and Nachwuchsförderung AO Trauma Deutschland (R.S.).

## Conflict of Interest

The authors declare that the research was conducted in the absence of any commercial or financial relationships that could be construed as a potential conflict of interest.

## Supplementary Material

Supplementary Table 1 Calculated absolute monocyte numbers and their subsets.

## Abbreviations

BSA, bovine serum albumin; Bcl-3, B-cell lymphoma 3-encoded protein; CD, cluster of differentiation; EDTA, ethylenediaminetetraacetic acid; FBS, fetal bovine serum; HV, healthy volunteers; IL, interleukin; LPS, lipopolysaccharide; MCP, monocyte chemoattractant protein; NLRP3, NLR family pyrin domain containing 3; NF-κB, nuclear factor ‘kappa-light-chain-enhancer’ of activated B-cells; PBS, phosphate-buffered saline; TLR, Toll-like receptor; TNF, tumor necrosis factor.

## References

[1] Szabo G Saha B. Alcohol’s Effect on Host Defense. Alcohol Res (2015) 37:159–70. 10.35946/arcr.v37.2.01 PMC4590613 26695755

[2] Piano MR. Alcohol’s Effects on the Cardiovascular System. Alcohol Res (2017) 38:219–41. 10.35946/arcr.v38.2.06 PMC5513687 28988575

[3] Gomes de Matos E Atzendorf J Kraus L Piontek D. Substanzkonsum in Der Allgemeinbevölkerung in Deutschland. Sucht (2016) 62:271–81. 10.1024/0939-5911/a000445

[4] John U Hanke M. Alcohol-Attributable Mortality in a High Per Capita Consumption Country – Germany. Alcohol Alcohol (2002) 37:581–5. 10.1093/alcalc/37.6.581 12414551

[5] Hadfield RJ Mercer M Parr MJ. Alcohol and Drug Abuse in Trauma. Resuscitation (2001) 48:25–36. 10.1016/s0300-9572(00)00315-4 11162880

[6] Hildebrand F Pape HC van Griensven M Meier S Hasenkamp S Krettek C. Genetic Predisposition for a Compromised Immune System After Multiple Trauma. Shock (2005) 24:518–22. 10.1097/01.shk.0000184212.97488.4e 16317381

[7] Afshar M Richards S Mann D Cross A Smith GB Netzer G. Acute Immunomodulatory Effects of Binge Alcohol Ingestion. Alcohol (2015) 49:57–64. 10.1016/j.alcohol.2014.10.002 25572859 PMC4314366

[8] Goral J Karavitis J Kovacs EJ. Exposure-Dependent Effects of Ethanol on the Innate Immune System. Alcohol (2008) 42:237–47. 10.1016/j.alcohol.2008.02.003 PMC2453223 18411007

[9] Pruett BS Pruett SB. An Explanation for the Paradoxical Induction and Suppression of an Acute Phase Response by Ethanol. Alcohol (2006) 39:105–10. 10.1016/j.alcohol.2006.08.003 PMC1764540 17134663

[10] Relja B Menke J Wagner N Auner B Voth M Nau C. Effects of Positive Blood Alcohol Concentration on Outcome and Systemic Interleukin-6 in Major Trauma Patients. Injury (2016) 47:640–5. 10.1016/j.injury.2016.01.016 26850862

[11] Sureshchandra S Raus A Jankeel A Ligh BJK Walter NAR Newman N. Dose-Dependent Effects of Chronic Alcohol Drinking on Peripheral Immune Responses. Sci Rep (2019) 9:7847. 10.1038/s41598-019-44302-3 31127176 PMC6534547

[12] Neupane SP Skulberg A Skulberg KR Aass HC Bramness JG. Cytokine Changes Following Acute Ethanol Intoxication in Healthy Men: A Crossover Study. Mediators Inflammation (2016) 2016:3758590. 10.1155/2016/3758590 PMC5206441 28090151

[13] Muralidharan S Lim A Catalano D Mandrekar P. Human Binge Alcohol Intake Inhibits TLR4-MyD88 and TLR4-TRIF Responses But Not the TLR3-TRIF Pathway: HspA1A and PP1 Play Selective Regulatory Roles. J Immunol (2018) 200:2291–303. 10.4049/jimmunol.1600924 PMC5860983 29445009

[14] Mandrekar P Catalano D White B Szabo G. Moderate Alcohol Intake in Humans Attenuates Monocyte Inflammatory Responses: Inhibition of Nuclear Regulatory Factor Kappa B and Induction of Interleukin 10. Alcohol Clin Exp Res (2006) 30:135–9. 10.1111/j.1530-0277.2006.00012.x 16433741

[15] Kapellos TS Bonaguro L Gemund I Reusch N Saglam A Hinkley ER. Human Monocyte Subsets and Phenotypes in Major Chronic Inflammatory Diseases. Front Immunol (2019) 10:2035. 10.3389/fimmu.2019.02035 31543877 PMC6728754

[16] Greco M Mazzei A Palumbo C Verri T Lobreglio G. Flow Cytometric Analysis of Monocytes Polarization and Reprogramming From Inflammatory to Immunosuppressive Phase During Sepsis. EJIFCC (2019) 30:371–84. PMC6893894 31814812

[17] Donnadieu-Rigole H Mura T Portales P Duroux-Richard I Bouthier M Eliaou JF. Effects of Alcohol Withdrawal on Monocyte Subset Defects in Chronic Alcohol Users. J Leukoc Biol (2016) 100:1191–9. 10.1189/jlb.5A0216-060RR 27256567

[18] von Elm E Altman DG Egger M Pocock SJ Gotzsche PC Vandenbroucke JP. The Strengthening the Reporting of Observational Studies in Epidemiology (STROBE) Statement: Guidelines for Reporting Observational Studies. J Clin Epidemiol (2008) 61:344–9. 10.1016/j.jclinepi.2007.11.008 18313558

[19] Sturm R Haag F Janicova A Xu B Vollrath JT Bundkirchen K. Acute alcohol consumption increases systemic endotoxin bioactivity for days in healthy volunteers-with reduced intestinal barrier loss in female. Eur J Trauma Emerg Surg (2021). 10.1007/s00068-021-01666-4 PMC9192383 33839799

[20] Wagner N Dieteren S Franz N Kohler K Perl M Marzi I. Alcoholinduced Attenuation of Posttraumatic Inflammation is Not Necessarily Liverprotective Following Trauma/Hemorrhage. Int J Mol Med (2019) 44:1127–38. 10.3892/ijmm.2019.4259 31257463

[21] Franz N Dieteren S Kohler K Mors K Sturm R Marzi I. Alcohol Binge Reduces Systemic Leukocyte Activation and Pulmonary Pmn Infiltration After Blunt Chest Trauma and Hemorrhagic Shock. Inflammation (2019) 42:690–701. 10.1007/s10753-018-0927-z 30411212

[22] Horauf JA Kany S Janicova A Xu B Vrdoljak T Sturm R. Short Exposure to Ethanol Diminishes Caspase-1 and ASC Activation in Human Hepg2 Cells In Vitro. Int J Mol Sci (2020) 21:3196. 10.3390/ijms21093196 PMC7246869 32366053

[23] Janicova A Becker N Xu B Wutzler S Vollrath JT Hildebrand F. Endogenous Uteroglobin as Intrinsic Anti-Inflammatory Signal Modulates Monocyte and Macrophage Subsets Distribution Upon Sepsis Induced Lung Injury. Front Immunol (2019) 10:2276. 10.3389/fimmu.2019.02276 31632392 PMC6779999

[24] Relja B Omid N Schaible A Perl M Meier S Oppermann E. Pre- or Post-Treatment With Ethanol and Ethyl Pyruvate Results in Distinct Anti-Inflammatory Responses of Human Lung Epithelial Cells Triggered by Interleukin-6. Mol Med Rep (2015) 12:2991–8. 10.3892/mmr.2015.3764 25954992

[25] Haag F Janicova A Xu B Powerski M Fachet M Bundkirchen K. Reduced Phagocytosis, ROS Production and Enhanced Apoptosis of Leukocytes Upon Alcohol Drinking in Healthy Volunteers. Eur J Trauma Emerg Surg (2021). 10.1007/s00068-021-01643-x PMC9360092 33783566

[26] Burnham EL Moss M Martin GS. The Effect of Alcohol Consumption on Risk for Sepsis and ARDS. In: JL Vincent (eds). Intensive Care Medicine. New York, NY: Springer (2003). 10.1007/978-1-4757-5548-0_9

[27] Kratofil RM Kubes P Deniset JF. Monocyte Conversion During Inflammation and Injury. Arterioscler Thromb Vasc Biol (2017) 37:35–42. 10.1161/ATVBAHA.116.308198 27765768

[28] Ren Z Wang X Xu M Yang F Frank JA Ke ZJ. Binge Ethanol Exposure Causes Endoplasmic Reticulum Stress, Oxidative Stress and Tissue Injury in the Pancreas. Oncotarget (2016) 7:54303–16. 10.18632/oncotarget.11103 PMC5342343 27527870

[29] Zhang K Wang H Xu M Frank JA Luo J. Role of MCP-1 and CCR2 in Ethanol-Induced Neuroinflammation and Neurodegeneration in the Developing Brain. J Neuroinflamm (2018) 15:197. 10.1186/s12974-018-1241-2 PMC6034273 29976212

[30] Gacouin A Roussel M Le Priol J Azzaoui I Uhel F Fest T. Acute Alcohol Exposure has an Independent Impact on C-reactive Protein Levels, Neutrophil CD64 Expression, and Subsets of Circulating White Blood Cells Differentiated by Flow Cytometry in Nontrauma Patients. Shock (2014) 42:192–8. 10.1097/SHK.0000000000000195 24827394

[31] Anton M Rodriguez-Gonzalez A Ballesta A Gonzalez N Del Pozo A de Fonseca FR. Alcohol Binge Disrupts the Rat Intestinal Barrier: The Partial Protective Role of Oleoylethanolamide. Br J Pharmacol (2018) 175:4464–79. 10.1111/bph.14501 PMC6255955 30248186

[32] Li X Wang C Nie J Lv D Wang T Xu Y. Toll-Like Receptor 4 Increases Intestinal Permeability Through Up-Regulation of Membrane PKC Activity in Alcoholic Steatohepatitis. Alcohol (2013) 47:459–65. 10.1016/j.alcohol.2013.05.004 23871536

[33] Liangpunsakul S Toh E Ross RA Heathers LE Chandler K Oshodi A. Quantity of Alcohol Drinking Positively Correlates With Serum Levels of Endotoxin and Markers of Monocyte Activation. Sci Rep (2017) 7:4462. 10.1038/s41598-017-04669-7 28667254 PMC5493657

[34] Bala S Tang A Catalano D Petrasek J Taha O Kodys K. Induction of Bcl-3 by Acute Binge Alcohol Results in Toll-Like Receptor 4/LPS Tolerance. J Leukoc Biol (2012) 92:611–20. 10.1189/jlb.0112050 PMC3427604 22782967

[35] Carmody RJ Ruan Q Palmer S Hilliard B Chen YH. Negative Regulation of Toll-Like Receptor Signaling by NF-kappaB p50 Ubiquitination Blockade. Science (2007) 317:675–8. 10.1126/science.1142953 17673665

[36] Eder C. Mechanisms of interleukin-1beta Release. Immunobiology (2009) 214:543–53. 10.1016/j.imbio.2008.11.007 19250700

[37] Bakele M Joos M Burdi S Allgaier N Poschel S Fehrenbacher B. Localization and Functionality of the Inflammasome in Neutrophils. J Biol Chem (2014) 289:5320–9. 10.1074/jbc.M113.505636 PMC3931087 24398679

[38] Netea MG van de Veerdonk FL van der Meer JW Dinarello CA Joosten LA. Inflammasome-Independent Regulation of IL-1-family Cytokines. Annu Rev Immunol (2015) 33:49–77. 10.1146/annurev-immunol-032414-112306 25493334

[39] Provoost S Maes T Pauwels NS Vanden Berghe T Vandenabeele P Lambrecht BN. NLRP3/Caspase-1-Independent IL-1beta Production Mediates Diesel Exhaust Particle-Induced Pulmonary Inflammation. J Immunol (2011) 187:3331–7. 10.4049/jimmunol.1004062 21844393

[40] Netea MG Joosten LA Latz E Mills KH Natoli G Stunnenberg HG. Trained Immunity: A Program of Innate Immune Memory in Health and Disease. Science (2016) 352:aaf1098. 10.1126/science.aaf1098 27102489 PMC5087274

[41] Mors K Horauf JA Kany S Wagner N Sturm R Woschek M. Ethanol Decreases Inflammatory Response in Human Lung Epithelial Cells by Inhibiting the Canonical NF-Kb-Pathway. Cell Physiol Biochem (2017) 43:17–30. 10.1159/000480313 28848184

[42] Otte JM Cario E Podolsky DK. Mechanisms of Cross Hyporesponsiveness to Toll-like Receptor Bacterial Ligands in Intestinal Epithelial Cells. Gastroenterology (2004) 126:1054–70. 10.1053/j.gastro.2004.01.007 15057745

[43] Ferrero ME Bertelli AE Fulgenzi A Pellegatta F Corsi MM Bonfrate M. Activity In Vitro of Resveratrol on Granulocyte and Monocyte Adhesion to Endothelium. Am J Clin Nutr (1998) 68:1208–14. 10.1093/ajcn/68.6.1208 9846848

[44] Duthie GG Pedersen MW Gardner PT Morrice PC Jenkinson AM McPhail DB. The Effect of Whisky and Wine Consumption on Total Phenol Content and Antioxidant Capacity of Plasma From Healthy Volunteers. Eur J Clin Nutr (1998) 52:733–6. 10.1038/sj.ejcn.1600635 9805220

[45] Imhof A Blagieva R Marx N Koenig W. Drinking Modulates Monocyte Migration in Healthy Subjects: A Randomised Intervention Study of Water, Ethanol, Red Wine and Beer With or Without Alcohol. Diabetes Vasc Dis Res (2008) 5:48–53. 10.3132/dvdr.2008.009 18398813

[46] Dasu MR Devaraj S Jialal I. High Glucose Induces IL-1beta Expression in Human Monocytes: Mechanistic Insights. Am J Physiol Endocrinol Metab (2007) 293:E337–46. 10.1152/ajpendo.00718.2006 PMC2676171 17426109

[47] Nandy D Janardhanan R Mukhopadhyay D Basu A. Effect of Hyperglycemia on Human Monocyte Activation. J Investig Med (2011) 59:661–7. 10.2310/JIM.0b013e31820ee432 PMC3143266 21307779

[48] Awad F Assrawi E Jumeau C Georgin-Lavialle S Cobret L Duquesnoy P. Impact of Human Monocyte and Macrophage Polarization on NLR Expression and NLRP3 Inflammasome Activation. PloS One (2017) 12:e0175336. 10.1371/journal.pone.0175336 28403163 PMC5389804

[49] Lage SL Dominical VM Wong CS Sereti I. Evaluation of Canonical Inflammasome Activation in Human Monocytes by Imaging Flow Cytometry. Front Immunol (2019) 10:1284. 10.3389/fimmu.2019.01284 31214205 PMC6558012

[50] Dayer JM. How T-lymphocytes are Activated and Become Activators by Cell-Cell Interaction. Eur Respir J Suppl (2003) 44:10s–5s. 10.1183/09031936.03.00000403b 14582893

[51] Kral JB Schrottmaier WC Salzmann M Assinger A. Platelet Interaction With Innate Immune Cells. Transfus Med Hemother (2016) 43:78–88. 10.1159/000444807 27226790 PMC4872052

[52] Lin SJ Chao HC Yan DC Huang YJ. Expression of Adhesion Molecules on T Lymphocytes in Young Children and Infants–a Comparative Study Using Whole Blood Lysis or Density Gradient Separation. Clin Lab Haematol (2002) 24:353–9. 10.1046/j.1365-2257.2002.00462.x 12452816

[53] Kany S Horstmann JP Sturm R Mors K Relja B. Reduced NLRP3 Gene Expression Limits the IL-1beta Cleavage Via Inflammasome in Monocytes From Severely Injured Trauma Patients. Mediators Inflammation (2018) 2018:1752836. 10.1155/2018/1752836 PMC5971319 29861655

[54] West SD Mold C. Monocyte Deactivation Correlates With Injury Severity Score, But Not With Heme Oxygenase-1 Levels in Trauma Patients. J Surg Res (2012) 172:5–10. 10.1016/j.jss.2011.04.016 21601878 PMC3191279

