# Supplementary material for: Acute Alcohol Intoxication Modulates Monocyte Subsets and Their Functions in a Time-Dependent Manner in Healthy Volunteers
Source: Front Immunol. 2021 May 18;12:652488. doi: 10.3389/fimmu.2021.652488 (PMC8167072; doi:10.3389/fimmu.2021.652488)
Supplement: Supplementary Table 1 — Calculated absolute monocyte numbers and their subsets. [file Table_1.docx]

|  | Cells per µL (mean ± SEM) | | | |
| --- | --- | --- | --- | --- |
| Timepoints | Monocytes/µL | CD14^bright^CD16^-^ monocytes | CD14^bright^CD16^+^ monocytes | CD14^dim^CD16^+^ monocytes |
| T0 | 409.85 ± 28.06 | 323.12 ± 24.00 | 19.93 ± 3.73 | 13.81 ± 1.40 |
| T2 | 400.98 ± 21.38 | 344.59 ± 17.72 | 27.84 ± 3.67 | 17.21 ± 1.91 |
| T4 | 492.04 ± 46.77 | 409.24 ± 39.42 | 32.76 ± 4.79 | 29.53 ± 3.41 |
| T6 | 369.56 ± 23.97 | 310.19 ± 19.76 | 25.64 ± 3.73 | 20.20 ± 2.05 |
| T24 | 520.05 ± 41.28 | 449.21 ± 34.90 | 24.08 ± 3.98 | 19.51 ± 2.73 |
| T48 | 421.35 ± 32.02 | 341.10 ± 25.89 | 19.56 ± 3.61 | 16.82 ± 3.33 |
